# Endoscopic Management of a Proximally Migrated Fully Covered SEMS Using the Stent-in-Stent Technique

**DOI:** 10.1155/2020/3438469

**Published:** 2020-03-26

**Authors:** Arunchai Chang, Varayu Prachayakul

**Affiliations:** ^1^Division of Gastroenterology, Department of Internal Medicine, Hatyai Hospital, Songkhla, Thailand; ^2^Siriraj Gastrointestinal Endoscopy Center, Division of Gastroenterology, Department of Internal Medicine, Siriraj Hospital, Faculty of Medicine, Mahidol University, Bangkok, Thailand

## Abstract

Endoscopic biliary decompression *via* stent placement is an important approach for the palliative management of distal malignant biliary obstruction. However, migration of the inserted stent can occur, either distally or proximally; proximal migration is less common, but it also presents a greater challenge for endoscopic resolution. We present a case of a 67-year-old woman who had locally advanced pancreatic cancer and developed a common bile duct obstruction. Upon clinical presentation of chronic, painless, progressive jaundice, the obstruction was managed by placing of a 10 mm × 60 mm covered self-expandable metal stent (CSEMS), which successfully facilitated palliative biliary drainage. Six months later, however, the patient developed recurrent jaundice, which was determined to be due to proximal migration of the CSEMS. Repeat endoscopic retrograde cholangiography was performed, and initial attempts to retrieve the migrated stent failed. Finally, another 10 mm × 60 mm CSEMS was placed across the stricture site, inside the previous stent, which remained in place. The treatment resolved the obstruction and jaundice, and the patient experienced no adverse events.

## 1. Introduction

Endoscopic biliary decompression *via* stent placement is an important modality for the palliative management of distal malignant biliary obstruction. The demonstrated longer stent patency of self-expandable metal stents (SEMSs), over that of the plastic-type stent, underlies the physicians' preference for their use, especially for cases in which the patient's survival is expected to be longer than 3 months [1, 2]. Many different designs of SEMSs are available, and in use, worldwide; these include uncovered, partially covered, and fully covered SEMSs. The choice of the specific SEMS type to be used is made on a case-by-case basis, usually by the endoscopist, based on the biliary pathological characteristics of the patients, including etiology, location of the obstruction, and institutional availability of the various SEMS types. The potential of SEMS malfunction is related to postplacement events, including stent migration or occlusion, and SEMSs can ever lead to life-threatening complications, such as stent-induced ulceration, duodenal obstruction, and perforation [3, 4]. For stent migration, many studies have demonstrated that covered SEMSs (CSEMSs) migrate more frequently than uncovered SEMSs (USEMSs) [5]. To resolve this issue, endoscopic techniques, using foreign body forceps, biopsy forceps, or polypectomy snares, have been used for stent retrieval. Stents can move either distally or proximally, and while proximal movement occurs less frequently, it presents a more difficult situation for endoscopic removal.

Here, we report an alternative endoscopic approach for the management of proximally migrated CSEMSs that was successfully applied in a case of malignant distal common bile duct obstruction, in which the endoscopist had failed to retrieve the stent; placement of another CSEMS was performed distally using a stent-in-stent technique after navigating through the previous CSEMS with a guidewire.

## 2. Case Report

**Figure 1:**
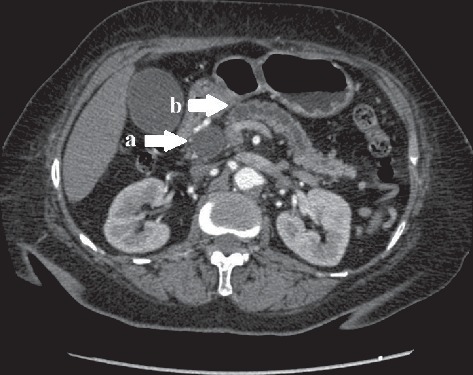
Computed tomography scan showing an ill-defined, hypodense mass at the pancreatic head, causing upstream dilatation of the common bile duct (a) and pancreatic duct (b) compatible with the double duct sign.

A 67-year-old woman presented to our department with a complaint of a 3-week history of progressive painless jaundice. Laboratory tests revealed no leukocytosis but mild anemia (hemoglobin: 11.5 g/dL, reference range: 12.0–18.0 g/dL). The liver chemistry panel revealed the following: aspartate aminotransferase: 38 U/L (reference range: 0–32 U/L); alanine aminotransferase: 31 U/L (reference range: 0–33 U/L); total bilirubin: 18.8 mg/dL (reference range: 0.0–1.2 mg/dL); direct bilirubin: 16.4 mg/dL (reference range: 0.0–0.3 mg/dL); alkaline phosphatase: 252 U/L (reference range: 35–105 U/L); and carbohydrate antigen 19–9: <0.6 U/mL (reference range: 0–39 U/mL). A computed tomography scan demonstrated an ill-defined mass at the pancreatic head (4.0 cm × 4.2 cm in size) causing distal common bile duct and pancreatic duct dilatation (Figure 1). The tumor encased the superior mesenteric vein and multiple intra-abdominal lymphadenopathies were also apparent.

**Figure 2:**
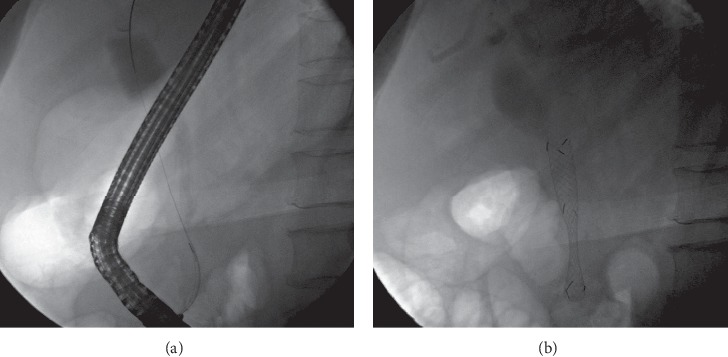
Cholangiography showing a long-segment stricture at the distal common bile duct. (a) The stricture caused upstream dilatation of the distal common bile duct; (b) after the placement of a fully covered self-expandable metal stent.

After the cytological assessment (using endoscopic ultrasonography with fine-needle aspiration) indicated adenocarcinoma, a locally advanced pancreatic malignancy was diagnosed. Endoscopic retrograde cholangiography (ERC) was subsequently performed and showed a long-segment stricture at the distal common bile duct that caused upstream dilatation (1.5 cm in diameter, together with dilations of the bilateral intrahepatic ducts; Figure 2(a)). The obstructive jaundice was resolved by placing a 10 mm × 60 mm fully covered SEMS (Niti-S Biliary Covered Stent™; TaeWoong Medical Co, Ltd., Gimposi, Gyeonggi-do, Korea), which facilitated biliary drainage (Figure 2(b)). The patient received palliative chemotherapy, consisting of cycles of a cisplatin and gemcitabine regimen.

**Figure 3:**
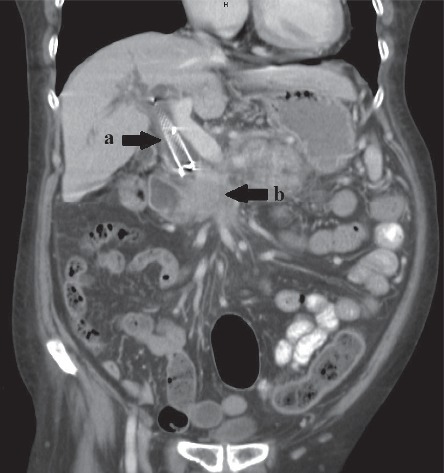
Computed tomography scan performed after the patient developed recurrent obstructive jaundice showing proximal migration of the previously placed metallic stent (a). The distal end of the stent was located above the pancreatic mass (b), and upstream dilatation of the common bile duct was apparent.

**Figure 4:**
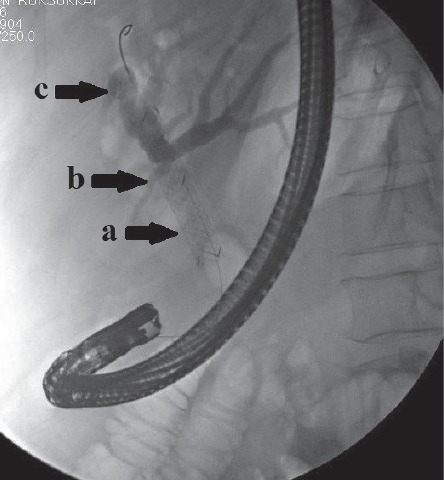
Cholangiography showing proximal migration of the previously placed SEMS. Upstream dilatation of the common bile duct, the common hepatic duct, and bilateral intrahepatic ducts are shown by (a), (b), and (c), respectively.

**Figure 5:**
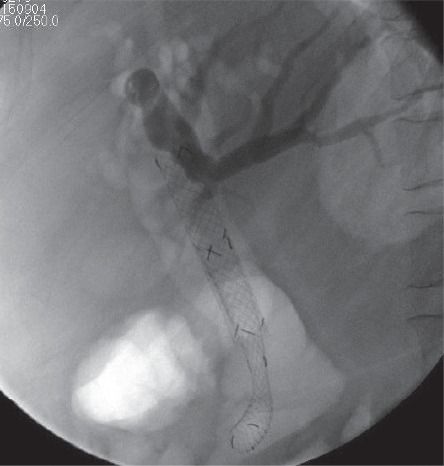
Fluoroscopic view of the stent-in-stent technique applied during the procedure.

Six months later, the patient presented with complaints of recurrent jaundice and was referred for ERC. A computed tomography scan demonstrated proximal migration of the previously placed CSEMS (Figure 3), and the patient was scheduled for endoscopic intervention. In the endoscopy suite, the cholangiogram verified proximal migration and showed upstream dilation of the common bile duct (Figure 4). The initial attempts to retrieve the migrated stent endoscopically by using rat tooth grasping forceps (Rat Tooth Alligator Jaw Grasping Forceps; Olympus Medical System Corporation, Aomori, Japan) and snares (Captivator™ Single-Use Snare, Boston Scientific Corporation, Costa Rica) failed. As a result, deep cannulation using a sphincterotome (Ultratome™ XL Triple Lumen; Boston Scientific Corporation, Costa Rica) was performed by navigating a 0.035-inch guidewire (Jagwire™, Boston Scientific Corporation, Costa Rica) until passage through the previous CSEMS was achieved proximally. A 12-mm balloon retrieval catheter (Extractor^TM^ Pro RX Balloon, Boston Scientific Limited, Ireland) was then inserted over the guidewire and inflated within the migrated stent to confirm proper location of the guidewire. Then, retrieval of the migrated stent was attempted by pulling the balloon back distally, but the attempt failed. Finally, an additional 10 cm × 60 mm CSEMS (Niti-S Biliary Covered Stent™; TaeWoong Medical Co, Ltd., Gimposi, Gyeonggi-do, Korea) was placed across the stricture site, inside the previous stent. Satisfactory drainage was achieved (Figure 5).

Following the procedure, the patient was hospitalized overnight (recovery was uneventful) and discharged to home the next morning. At the follow-up 2 weeks after the procedure, the bilirubin level was normal. The patient refused the recommended chemotherapy and passed away 5 months later without recurrent episodes of jaundice.

## 3. Discussion

The majority of malignant distal common bile duct strictures are caused by pancreaticobiliary cancers. It has been reported that up to 80% of patients with pancreatic carcinoma develop obstructive jaundice; unfortunately, most of these patients have advanced-stage disease at the time of diagnosis [6]. Endoscopic biliary drainage *via* biliary stenting is the most common approach for addressing the issue of obstructive jaundice and can improve the quality of life of the patient, regardless of the predicted cancer-related survival time [7]. SEMSs have gained popularity among treating physicians, as they have longer stent patency than plastic-type stents, which are prone to occlusion by microbacterial biofilm formation [1, 8].

Nevertheless, SEMSs harbor their own disadvantages; for example, USEMSs can be occluded due to tumor ingrowth through the mesh [5]. CSEMSs have better patency than USEMSs due to their thin, nonporous membrane, which is located inside of their mesh and prevents tumor ingrowth [9, 10]. The disadvantage of CSEMSs, however, is their lower axial force, which leads to an increased risk of migration [11]. Unfortunately, the multitude of studies comparing CSEMSs and USEMSs to determine which is superior for the treatment of malignancy distal biliary obstruction have yielded conflicting results [9, 10, 12, 13]. Adding to the controversy, a prospective randomized trial published in 2010 showed no significant difference in stent patency between CSEMSs and USEMSs for the palliative treatment of distal malignant biliary obstruction, but confirmed that tumor ingrowth was more common in USEMSs and that CSEMSs had an increased risk of stent migration [14].

Stent migration is a well-known clinical problem, affecting 1.8% to 11.5% of biliary stent patients [5, 9, 10, 12, 13]. When stented patients develop recurrent obstructive jaundice or cholangitis, migration should be among the first events suspected. Proximal migration is much less common than distal migration, reportedly affecting only 1.7% of partially covered SEMSs [15]. Regardless of the migration pattern, several techniques for retrieval have been described in the literature, including the use of foreign body forceps, biopsy forceps, polypectomy snares, or Dormia baskets [16–18].

In our patient, the initial attempt to remove the proximally migrated CSEMS was carried out with several of the commonly used accessories, all of which failed. Since the upper border of the migrated stent was still wide open, the endoscopist decided to abandon removal of the migrated stent and instead place another stent across the stricture point, using a stent-in-stent technique. No stent-related complications were observed in the patient following the procedure, including the most common complication, cholecystitis, which occurs in up to 10% of patients after SEMS placement and is associated with preexisting cystic duct lesions, such as cholelithiasis or tumor invasion of the cystic duct [19, 20].

## 4. Conclusion

The proximal migration of a fully covered self-expandable metal stent in patients with distal malignant biliary obstruction occurs less frequently than distal migration and presents a more difficult situation for endoscopic removal. The stent-in-stent technique is an alternative treatment approach in cases where endoscopist had failed to retrieve the stent.

## Abbreviations

SEMS: Self-expandable metal stent
CSEMS: Covered self-expandable metal stent
USEMS: Uncovered self-expandable metal stent
ERC: Endoscopic retrograde cholangiography
